# Risk of Ischemic and Hemorrhagic Stroke in Individuals With Type 1 and Type 2 Diabetes: A Nationwide Cohort Study in Sweden

**DOI:** 10.1212/WNL.0000000000213480

**Published:** 2025-03-13

**Authors:** Anastasios Mavridis, Adam Viktorisson, Björn Eliasson, Mia von Euler, Katharina S. Sunnerhagen

**Affiliations:** 1 Department of Clinical Neuroscience, Institute of Neuroscience and Physiology, Sahlgrenska Academy, University of Gothenburg, Sweden;; 2 The Sahlgrenska University Hospital, Gothenburg, Sweden;; 3 The National Diabetes Register, Center of Registers, Gothenburg, Sweden; and; 4 Department of Neurology and Rehabilitation, Faculty of Medicine and Health, Örebro University, Sweden.

## Abstract

**Background and Objectives:**

Diabetes significantly increases the risk of cardiovascular events, including stroke. Although the association with ischemic stroke is well established, the relationship with hemorrhagic stroke remains unclear. This study aimed to evaluate the risk of ischemic and hemorrhagic stroke in individuals with type 1 and type 2 diabetes compared with diabetes-free controls from the general population.

**Methods:**

This cohort study included individuals with type 1 or type 2 diabetes from the Swedish National Diabetes Register between 2005 and 2019, matched to diabetes-free controls by age and sex. Data on baseline characteristics, comorbidities, medications, and outcomes were collected from multiple national registers. Stroke incidence rates and adjusted hazard ratios were estimated using Cox proportional hazard models, stratified by diabetes type, for ischemic and hemorrhagic stroke.

**Results:**

The study included 47,720 individuals with type 1 diabetes (mean age 34.4, 44.8% female) and 686,158 with type 2 diabetes (mean age 65.3, 43.3% female), matched to 143,160 and 2,058,474 controls, respectively. In individuals with type 1 diabetes, the ischemic stroke risk was 2.54 times higher (95% CI 2.36–2.73) and the hemorrhagic stroke risk was 1.88 times higher (95% CI 1.57–2.26) compared with controls. In individuals with type 2 diabetes, the ischemic stroke risk was 1.37 times higher (95% CI 1.35–1.38) while the hemorrhagic stroke risk was not significantly increased (HR: 0.99, 95% CI 0.96–1.02). Higher HbA1c levels were associated with increased ischemic stroke risk for both diabetes types. For hemorrhagic stroke, individuals with type 1 diabetes had significantly higher risk starting at HbA1c > 52 mmol/mol while in those with type 2 diabetes, a modest risk increase was observed only at HbA1c > 72 mmol/mol.

**Discussion:**

The risk of ischemic stroke was higher for both diabetes types. Individuals with type 1 diabetes also exhibited a higher risk of hemorrhagic stroke compared with diabetes-free controls while type 2 diabetes was significantly associated with risk of hemorrhagic stroke only when HbA1c was higher than 72 mmol/mol. These findings highlight the increased stroke risk in diabetes, with distinct patterns by stroke subtype and diabetes type. Tailored prevention strategies are essential to address these differences.

## Introduction

Diabetes mellitus is a multifaceted and chronic metabolic disease with complications regarding end-organ dysfunction or failure.^1^ The global burden of diabetes was estimated at 463 million cases, 1.55 million excess deaths, and 37 million disability-adjusted life years in 2019.^2,3^ The total disease burden has approximately doubled since the 1990s,^2^ and a continued increase in diabetes-related mortality rates is expected henceforth.^4^ Patients with diabetes are at risk of cardiovascular events, with 2–4 times higher morbidity and mortality compared with the general population.^5^ Overall, diabetes is concomitant in approximately one-third of patients with stroke and associated with a worse poststroke prognosis.^6^ The excess risk of stroke may, however, be prevented through ameliorating control of metabolic and behavioral risk factors.^7^

While diabetes is known to increase the risk of ischemic stroke by 1.5–2 times, its impact on hemorrhagic stroke risk is less clear and has not been extensively studied.^8^ Findings from various studies are contradictory,^9^ with some suggesting that diabetes is associated with a lower risk of hemorrhagic stroke,^10^ others finding no association,^11^ and some indicating a higher risk in people with diabetes.^12^

In this study, we investigate the risk of ischemic and hemorrhagic stroke in individuals with type 1 and type 2 diabetes compared with matched diabetes-free controls from the general population.

## Methods

### Standard Protocol Approvals, Registrations, and Patient Consents

The study was approved by the Swedish Ethical Review Authority on August 18th, 2021 (registration number: 2021–03645). Individual consent is not required to report patients to national quality registries of health care according to Swedish law (Patient Data Act 2008:355, chapter 7).

### Data Sources

Established in 1996, the National Diabetes Register (NDR) includes information on diabetes care, risk factors, and complications from most of the persons with diabetes at 18 years of age or older in Sweden.^13^ All individuals with a diabetes diagnosis in the NDR between January 1, 2005, and December 31, 2019, were included. Each patient with diabetes was matched with 3 randomly selected diabetes-free controls from the general population using exact matching on age and sex. The matched controls were selected from the Total Population Register in Sweden^14^ on the date of first registration in the NDR.

Information on incident stroke events and comorbid conditions was collected from the National Patient Register,^15^ which comprises data from inpatient (hospital) and outpatient specialist care in Sweden. Mortality rates and causes of death were collected from the Swedish Cause of Death Register.^16^ Information on medical treatments was collected from the National Prescribed Drug Register.^17^ Information on education level and income was collected from the longitudinal integrated database for health insurance and labor market studies (LISA).^18^ All the abovementioned registers were used for the collection of data for both individuals with diabetes and diabetes-free controls. Data linkage was performed by Statistics Sweden and the National Board of Health and Welfare using the personal identity numbers that are provided to all residents in Sweden at birth or shortly after immigration, and pseudonymized data were given to the researchers.

### Diabetes Diagnoses

Epidemiologic criteria were applied to ascertain diabetes diagnoses. Type 1 diabetes group was defined as having been diagnosed at 30 years of age or younger and being treated with insulin. Type 2 diabetes group was defined as having been diagnosed at any age and being treated with diet alone, or with the use of oral antihyperglycemic agents, or with insulin (with or without oral antihyperglycemic agents) if diagnosed at an age of 40 years or older. In Sweden, specialist clinics manage patients with type 1 diabetes while primary health care providers typically manage patients with type 2 diabetes.

### Variables

#### Sociodemographic Characteristics

Baseline sociodemographic characteristics included age, sex, education level of the previous year (categorized into 3 levels as lower: ≤9 years, middle: 10–12 years, higher: >12 years), and family income of the previous year (in hundreds of Swedish Krona and categorized into 3 equal tertiles for type 1 diabetes [lower: 0–2,221; middle: 2,222–4,145; higher: >4,145] and for type 2 diabetes [lower: 0–2030; middle: 2031–3,745; higher: >3,745]). Baseline smoking status was collected from the first NDR registration only for individuals with diabetes.

#### Comorbidities

Baseline comorbidities were identified from inpatient and outpatient diagnoses recorded for each patient from 4 years to 1 day before baseline, according to the International Classification of Diseases, 10th revision (ICD-10). The included conditions were cancer (C00-C96), dementia (F01-F03), alcohol or substance abuse (F10-F19), hypertension (I10-I15), atrial fibrillation (I48), myocardial infarction (I21-I23), heart failure (I50), TIA (G45.9), and previous stroke (ischemic [I63], hemorrhagic [I61], or unspecified type [I64]).

#### Medications

Baseline medications included all prescriptions collected from pharmacies within 6 months before and 6 months after baseline registration. The included medications were antiplatelet agents, anticoagulants, antihypertensives, diuretics, lipid-lowering medications, and β-blockers/antiarrhythmics/glycosides.

#### Laboratory Values

Baseline laboratory values were collected from the first NDR registration only for individuals with diabetes. The included values were low-density lipoprotein (LDL), systolic and diastolic blood pressure (BP), estimated glomerular filtration rate (eGFR), and body mass index (BMI).

#### Glycemic Control

Baseline glycated hemoglobin (HbA1c) was collected from the first NDR registration, and individuals with diabetes were classified into 5 categories based on their HbA1c levels: ≤42, 42–52, 52–62, 62–72, and >72 mmol/mol. HbA1c reflects the average blood glucose levels over the past 2 to 3 months and is a widely used indicator of long-term diabetes management.

### Outcomes

The primary outcome was time to the first ischemic or hemorrhagic stroke occurrence, measured from the date of diabetes diagnosis in the NDR. Incident stroke rates were followed until death or censoring on December 31, 2022. If both types of stroke occurred, only the first event was taken into consideration. The follow-up time for each patient and their corresponding matched controls was initiated on the same date. Stroke diagnoses were defined according to the ICD-10, encompassing ischemic stroke (I63) and hemorrhagic stroke (I61). Strokes of unspecified type (I64) were classified as ischemic strokes, based on Swedish stroke guidelines, which recommend that all patients with stroke undergo emergency CT scan to rule out hemorrhage.

### Statistical Analyses

#### Descriptive Characteristics

Baseline descriptive characteristics were presented using means and SDs for continuous variables or counts and valid percentages for categorical variables.

#### Stroke Risk and Incidence

Unadjusted risk ratios (RRs) with 95% CIs between individuals with diabetes and diabetes-free controls were calculated for ischemic stroke and hemorrhagic stroke for both diabetes types. The annual incidence rates (per 1,000 person-years) of ischemic and hemorrhagic stroke in individuals with diabetes and diabetes-free controls were calculated for each year between 2007 and 2022. Scarcity of data in the years 2005–2007 did not allow for accurate calculation of incidence because it led to overestimation in both groups; hence, these years were excluded.

The crude cumulative incidence of ischemic and hemorrhagic stroke in individuals with diabetes vs diabetes-free controls was graphically depicted using Kaplan-Meier curves, with 95% CIs.

Cox proportional hazard (PH) models were used to estimate the adjusted hazard of ischemic and hemorrhagic strokes separately for individuals with type 1 and type 2 diabetes. In each model, only 1 type of stroke was treated as the primary event. For the ischemic stroke model, individuals who experienced a hemorrhagic stroke or who died before having an ischemic stroke were censored at the time of that event. For the hemorrhagic stroke model, individuals who experienced an ischemic stroke or who died before having a hemorrhagic stroke were censored at the time of that event. Time to event was calculated from baseline until the first occurrence of the specified stroke, death, occurrence of another stroke type, or censoring at the end of the study on December 31, 2022. Covariates inserted in the model were age, sex, diabetes, education and income level, comorbidities, and medications.

The PH assumption was evaluated visually using scaled Schoenfeld residuals plots (eFigures 1–4). For all models, variables seemed to satisfy the PH assumption, except for previous stroke, which showed a stronger impact during the first years. Despite this deviation, previous stroke was retained in the models because of the substantial improvement in model performance, the consistent direction of its effect over time, and its clinical significance as a key predictor of stroke incidence.

#### Stratified Analysis by HbA1c Levels

Individuals with diabetes were categorized into 5 strata based on their baseline HbA1c levels. The matched diabetes-free controls were assigned to the same stratum as their corresponding participant with diabetes. Cox PH models of ischemic and hemorrhagic stroke were then fit for each HbA1c stratum, following the same methodology as described previously.

All statistical tests were 2-tailed and performed at α 5%. Statistical analyses were performed separately for type 1 and type 2 diabetes using R (R Core Team [2023], R: A Language and Environment for Statistical Computing, R Foundation for Statistical Computing, Vienna, Austria) and Statistical Package for the Social Sciences (SPSS) (IBM SPSS Statistics for Windows, Version 29.0, released 2022; IBM Corp., Armonk, NY).

### Data Availability

According to the Swedish regulations,^19^ data can only be used in accordance with the application for this study that is approved by the ethical board. Requests to access the data set can be submitted by qualified researchers to the authors (contact: Professor Katharina S. Sunnerhagen, email: ks.sunnerhagen@neuro.gu.se).

## Results

### Study Population

Between January 1, 2005, and December 31, 2019, a total of 733,878 individuals with type 1 or type 2 diabetes, identified through epidemiologic criteria in the NDR, were included in the study. Of those, 47,720 were diagnosed with type 1 diabetes and were matched to 143,160 diabetes-free controls, and 686,158 were diagnosed with type 2 diabetes and were matched to 2,058,474 diabetes-free controls. The total study sample consisted of 2,935,512 individuals.

### Baseline Characteristics

Baseline characteristics of individuals with type 1 and type 2 diabetes and their matched diabetes-free controls are provided in Table 1. The mean age of individuals with type 1 diabetes was 34.4 years, with 55.2% male. Among those with type 2 diabetes, the mean age was 65.3 years and 56.7% were male. Individuals with type 1 diabetes had higher rates of hypertension, myocardial infarction, heart failure, previous stroke, and alcohol or substance abuse compared with diabetes-free controls. Similarly, hypertension, atrial fibrillation, myocardial infarction, heart failure, and previous stroke were more common in those with type 2 diabetes compared with their diabetes-free controls. Baseline medication use was higher in individuals with type 2 diabetes compared with diabetes-free controls while for the type 1 diabetes group, similar or lower percentages of medication use were observed. Education and income levels were lower for both type 1 and type 2 diabetes groups relative to their matched diabetes-free controls. Differences between the 2 types of diabetes groups were evident across glycemic control, cardiovascular risk factors, and body composition. Individuals with type 1 diabetes had higher HbA1c and better eGFR while those with type 2 diabetes exhibited higher LDL levels, BP, and BMI.

**Table 1:** Baseline Characteristics of Individuals With Type 1 and Type 2 Diabetes and Diabetes-Free Controls

	Controls (type 1 diabetes), n = 143,160 (75.0%)	Type 1 diabetes, n = 47,720 (25.0%)	Controls (type 2 diabetes), n = 2,058,474 (75.0%)	Type 2 diabetes, n = 686,158 (25.0%)
Age, mean (SD)	34.4 (15.3)	34.4 (15.3)	65.3 (12.1)	65.3 (12.1)
Sex, male, n (%)	79,011 (55.2)	26,337 (55.2)	1,167,150 (56.7)	389,050 (56.7)
NDR information	NA	Mean (SD)	NA	Mean (SD)
HbA1c, mmol/mol	NA	66.1 (17.4)	NA	54.9 (16.8)
LDL, mmol/L	NA	2.6 (0.8)	NA	3.0 (1.0)
Systolic BP, mm Hg	NA	124.9 (16.1)	NA	138.4 (17.7)
Diastolic BP, mm Hg	NA	73.4 (9.2)	NA	79.2 (10.1)
eGFR, mL/minute/1.73 m^2^	NA	105.0 (30.4)	NA	82.2 (24.2)
BMI, kg/m^2^	NA	25.1 (4.4)	NA	30.2 (5.6)
Smoking, yes, n (%)	NA	6,147 (12.9)	NA	83,439 (12.2)
Country of birth	n (%)	n (%)	n (%)	n (%)
Sweden	116,395 (81.3)	43,128 (90.4)	1,685,247 (81.9)	546,207 (79.6)
Europe except Sweden	15,918 (11.1)	2,244 (4.7)	223,110 (10.8)	82,755 (12.1)
Other	10,825 (7.6)	2,346 (4.9)	149,769 (7.3)	57,181 (8.3)
Medications	n (%)	n (%)	n (%)	n (%)
Anticoagulants, yes	8,245 (5.8)	1,158 (2.4)	138,481 (6.7)	82,248 (12.0)
Antiplatelets, yes	24,845 (17.4)	7,133 (14.9)	377,513 (18.3)	258,384 (37.7)
Antihypertensives, yes	41,286 (28.8)	13,064 (27.4)	652,840 (31.7)	451,406 (65.8)
Diuretics, yes	22,040 (15.4)	5,194 (10.9)	331,392 (16.1)	229,603 (33.5)
Lipid-lowering drugs, yes	30,124 (21.0)	12,407 (26.0)	474,671 (23.1)	376,205 (54.8)
β-blockers or antiarrhythmics, yes	31,029 (21.7)	5,939 (12.4)	472,462 (23.0)	305,245 (44.5)
Comorbidities	n (%)	n (%)	n (%)	n (%)
Cancer, yes	2059 (1.4)	557 (1.2)	170,664 (8.3)	56,055 (8.2)
Dementia, yes	103 (0.1)	48 (0.1)	27,024 (1.3)	5,451 (0.8)
Alcohol or drug abuse, yes	2,491 (1.7)	1,412 (3.0)	36,089 (1.8)	18,218 (2.7)
Hypertension, yes	1764 (1.2)	4,704 (9.9)	231,066 (11.2)	169,687 (24.7)
Atrial fibrillation, yes	633 (0.4)	290 (0.6)	102,347 (5.0)	51,969 (7.6)
Myocardial infarction, yes	358 (0.3)	579 (1.2)	36,900 (1.8)	29,735 (4.3)
Heart failure, yes	318 (0.2)	576 (1.2)	55,535 (2.7)	38,896 (5.7)
Transient ischemic attack, yes	121 (0.1)	99 (0.2)	17,268 (0.8)	7,710 (1.1)
Previous stroke, yes	340 (0.2)	442 (0.9)	41,871 (2.0)	23,208 (3.4)
Education	n (%)	n (%)	n (%)	n (%)
Lower (≤9 y)	43,162 (31.2)	15,218 (33.1)	653,977 (32.2)	260,421 (39.0)
Middle (10–12 y)	59,238 (42.9)	20,453 (44.5)	836,215 (41.2)	283,577 (42.5)
Higher (>12 y)	35,746 (25.9)	10,282 (22.4)	538,530 (26.5)	122,906 (18.4)
Income (hundreds of Swedish Krona)	n (%)	n (%)	n (%)	n (%)
Lower	46,286 (32.6)	16,784 (35.7)	651,917 (31.7)	260,443 (38.2)
Middle	47,405 (33.3)	15,675 (33.3)	676,800 (32.9)	235,525 (34.6)
Higher	48,496 (34.1)	14,568 (31.0)	726,976 (35.4)	185,297 (27.2)
Outcomes	n (%)	n (%)	n (%)	n (%)
Ischemic stroke	2,110 (1.5)	1887 (4.0)	126,144 (6.1)	62,007 (9.0)
Hemorrhagic stroke	408 (0.3)	264 (0.6)	20,132 (1.0)	6,971 (1.0)

Abbreviations: BMI = body mass index; BP = blood pressure; eGFR = estimated glomerular filtration rate; HbA1c = glycated hemoglobin; LDL = low-density lipoprotein; NA = not available; NDR = National Diabetes Register.

All percentages are valid percentages.

Missing values (type 1) are as follows: HbA1c: 3,290; LDL: 29,299; systolic BP: 5,812; diastolic BP: 6,022; eGFR: 25,562; BMI: 11,233; smoking: 6,044; country: 24; education: 6,781; income: 1,666.

Missing values (type 2) are as follows: HbA1c: 69,396; LDL: 293,678; systolic BP: 97,344; diastolic BP: 98,652; eGFR: 170,761; BMI: 187,644; smoking: 162,167; country: 363; education: 49,006; income: 7,674.

### Stroke Risk and Incidence

In individuals with type 1 diabetes, the unadjusted risk ratio of ischemic stroke was 2.68 (2.52–2.85) and of hemorrhagic stroke was 1.94 (1.66–2.27) compared with diabetes-free controls. In those with type 2 diabetes, the risk ratio of ischemic stroke was 1.47 (1.46–1.49) while the risk ratio of hemorrhagic stroke showed only a modest increase at 1.04 (1.01–1.07), compared with diabetes-free controls.

**Figure 1:**
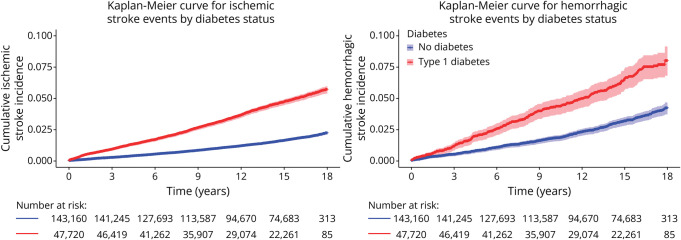
Cumulative Incidence of Ischemic and Hemorrhagic Stroke in Individuals With Type 1 Diabetes vs Diabetes-Free Controls

**Figure 2:**
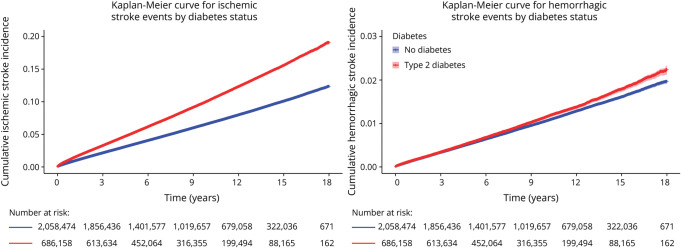
Cumulative Incidence of Ischemic and Hemorrhagic Stroke in Individuals With Type 2 Diabetes vs Diabetes-Free Controls

The crude cumulative incidence of stroke is shown in Figures 1 and 2 for individuals with type 1 and type 2 diabetes, respectively. Both ischemic and hemorrhagic strokes demonstrated a higher cumulative incidence among individuals with type 1 diabetes compared with the diabetes-free controls. In individuals with type 2 diabetes, the cumulative incidence of ischemic stroke was elevated relative to matched diabetes-free controls while the incidence curves for hemorrhagic stroke followed a similar trajectory between groups.

**Figure 3:**
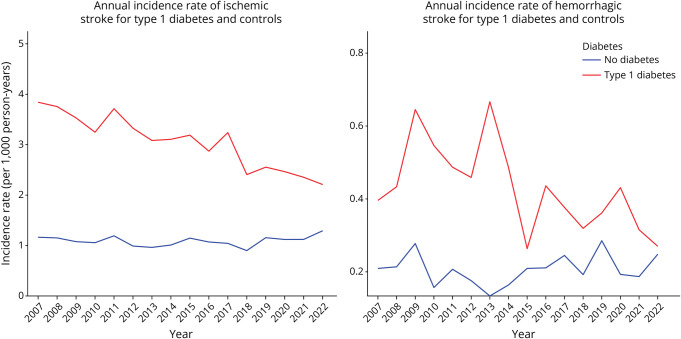
Annual Incidence Rates of Ischemic and Hemorrhagic Stroke in Individuals With Type 1 Diabetes and Diabetes-Free Controls (2007–2022)

**Figure 4:**
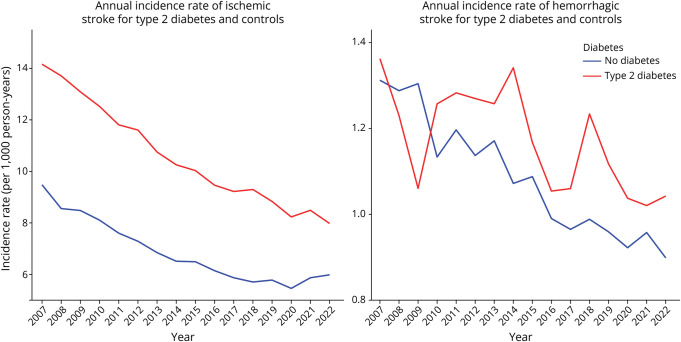
Annual Incidence Rates of Ischemic and Hemorrhagic Stroke in Individuals With Type 2 Diabetes and Diabetes-Free Controls (2007–2022)

The annual incidence rates for both stroke types are shown in Figures 3 and 4 for type 1 and type 2 diabetes. In individuals with type 1 diabetes, the annual incidence rate of ischemic stroke declined from 2007 to 2022 while the rates remained stable in the control group. Hemorrhagic stroke showed greater temporal variability, with a declining trend among individuals with type 1 diabetes, while a stable trend was observed in the diabetes-free control group. For type 2 diabetes, the incidence rate of ischemic stroke decreased consistently in both groups, with a more pronounced reduction in those with diabetes compared with diabetes-free controls. Hemorrhagic stroke rates in type 2 diabetes also showed a similar declining trend, characterized by high temporal variability in both groups.

#### Type 1 Diabetes

Cox PH regression results for individuals with type 1 diabetes and diabetes-free controls are presented in Table 2. For ischemic stroke, individuals with type 1 diabetes exhibited 2.5-fold higher risk compared with diabetes-free controls (hazard ratio [HR] = 2.54, 95% CI 2.36–2.73). Female sex and higher level of education and income decreased the risk of ischemic stroke. Older age; history of stroke; and several comorbidities, including alcohol or substance abuse, hypertension, atrial fibrillation, myocardial infarction, and heart failure, significantly increased ischemic stroke hazard. Individuals with type 1 diabetes had 88% higher risk of hemorrhagic stroke compared with diabetes-free controls (HR = 1.88, 95% CI 1.57–2.26). Female sex and higher education and income levels were significantly associated with lower risk of hemorrhagic stroke while older age, cancer, hypertension, and history of stroke were significantly associated with higher risk.

**Table 2:** Cox Regression Results for the Type 1 Diabetes Group and Diabetes-Free Controls

	Ischemic stroke		Hemorrhagic stroke	
Baseline covariates	*p* Value	HR (95% CI)	*p* Value	HR (95% CI)
Type 1 diabetes				
Diabetes (type 1, ref. control)	<0.001	2.54 (2.36–2.73)	<0.001	1.88 (1.57–2.26)
Age (18–88 y)	<0.001	1.08 (1.08–1.08)	<0.001	1.07 (1.06–1.07)
Sex, female (ref. male)	<0.001	0.74 (0.70–0.79)	<0.001	0.66 (0.56–0.77)
Anticoagulants, Yes	<0.001	1.27 (1.11–1.45)	0.533	1.12 (0.79–1.57)
Antiplatelets, yes	0.017	1.11 (1.02–1.21)	0.324	0.89 (0.72–1.12)
Antihypertensives, yes	0.460	1.03 (0.95–1.12)	0.623	1.05 (0.86–1.29)
Diuretics, yes	0.010	1.12 (1.03–1.22)	0.001	1.42 (1.15–1.76)
Lipid-lowering drugs, yes	0.370	0.96 (0.89–1.05)	0.070	0.82 (0.67–1.02)
β-blockers/antiarrhythmics, yes	0.905	1.00 (0.91–1.08)	0.353	1.11 (0.89–1.37)
Cancer, yes	0.309	0.92 (0.77–1.09)	0.010	1.58 (1.12–2.24)
Dementia, yes	0.172	1.46 (0.85–2.53)	0.798	0.77 (0.11–5.53)
Alcohol use, yes	<0.001	2.05 (1.71–2.46)	0.303	1.31 (0.78–2.21)
Hypertension, yes	<0.001	1.39 (1.27–1.52)	<0.001	1.80 (1.43–2.27)
Atrial fibrillation, yes	0.017	1.26 (1.04–1.52)	0.130	0.61 (0.32–1.16)
Myocardial infarction, yes	0.001	1.36 (1.13–1.64)	0.097	0.54 (0.26–1.12)
Heart failure, yes	0.002	1.36 (1.11–1.66)	0.166	1.48 (0.85–2.56)
Transient ischemic attack, yes	0.804	0.96 (0.69–1.33)	0.343	1.45 (0.67–3.16)
Previous stroke, yes	<0.001	5.63 (4.93–6.43)	<0.001	6.16 (4.37–8.68)
Income (ref. lower)	<0.001		<0.001	
Medium	<0.001	0.81 (0.76–0.87)	0.001	0.75 (0.63–0.89)
Higher	<0.001	0.66 (0.60–0.72)	<0.001	0.65 (0.52–0.81)
Education (ref. lower)	<0.001		0.004	
Medium	0.062	0.93 (0.87–1.00)	0.258	0.90 (0.75–1.08)
Higher	<0.001	0.75 (0.69–0.82)	<0.001	0.69 (0.55–0.86)
Type 2 diabetes				
Diabetes (type 2, ref. control)	<0.001	1.37 (1.35–1.38)	0.621	0.99 (0.96–1.02)
Age (18–85 y)	<0.001	1.07 (1.07–1.07)	<0.001	1.06 (1.06–1.06)
Sex, female (ref. male)	<0.001	0.75 (0.74–0.75)	<0.001	0.67 (0.66–0.69)
Anticoagulants, yes	0.483	1.01 (0.99–1.02)	<0.001	1.12 (1.07–1.18)
Antiplatelets, yes	<0.001	1.18 (1.17–1.19)	0.625	0.99 (0.96–1.03)
Antihypertensives, yes	<0.001	0.97 (0.96–0.98)	0.762	1.01 (0.97–1.04)
Diuretics, yes	0.941	1.00 (0.99–1.01)	0.689	0.99 (0.96–1.03)
Lipid-lowering drugs, yes	<0.001	0.96 (0.95–0.97)	<0.001	0.93 (0.90–0.96)
β-blockers/antiarrhythmics, yes	0.005	1.02 (1.01–1.03)	0.025	1.04 (1.01–1.07)
Cancer, yes	0.663	1.00 (0.99–1.02)	<0.001	1.09 (1.04–1.13)
Dementia, yes	0.079	0.96 (0.92–1.01)	<0.001	1.29 (1.16–1.44)
Alcohol use, yes	<0.001	1.57 (1.52–1.62)	<0.001	1.83 (1.70–1.98)
Hypertension, yes	<0.001	1.24 (1.22–1.25)	<0.001	1.37 (1.33–1.42)
Atrial fibrillation, yes	<0.001	1.29 (1.27–1.31)	<0.001	1.41 (1.35–1.47)
Myocardial infarction, yes	<0.001	1.07 (1.04–1.09)	0.005	0.90 (0.84–0.97)
Heart failure, yes	<0.001	1.14 (1.12–1.17)	0.021	0.93 (0.87–0.99)
Transient ischemic attack, yes	<0.001	1.45 (1.40–1.49)	<0.001	1.23 (1.12–1.34)
Previous stroke, yes	<0.001	3.04 (2.99–3.10)	<0.001	2.95 (2.81–3.10)
Income (ref. lower)	<0.001		<0.001	
Medium	<0.001	0.86 (0.85–0.87)	<0.001	0.90 (0.88–0.93)
Higher	<0.001	0.70 (0.69–0.71)	<0.001	0.73 (0.70–0.75)
Education (ref. lower)	<0.001		<0.001	
Medium	<0.001	0.96 (0.95–0.97)	0.348	0.99 (0.96–1.01)
Higher	<0.001	0.87 (0.86–0.88)	<0.001	0.92 (0.89–0.96)

Abbreviation: HR = hazard ratio.

Model fit ischemic-type 1: -2LL: 84,781, χ^2^: 18,734, *p* < 0.001; type 2: -2LL: 5,147,834, χ^2^: 179,462, *p* < 0.001.

Model fit hemorrhagic-type 1: -2LL: 14,436, χ^2^: 2,382, *p* < 0.001; type 2: -2LL: 746,394, χ^2^: 20,691, *p* < 0.001.

#### Type 2 Diabetes

Cox PH regression results for individuals with type 2 diabetes and diabetes-free controls are presented in Table 2. Individuals with type 2 diabetes had 37% higher risk of ischemic stroke compared with diabetes-free controls (HR = 1.37, 95% CI 1.35–1.38). Similar to type 1 diabetes, female sex and higher levels of education and income decreased the risk of ischemic stroke. With the exception of dementia and cancer, all comorbidities were significantly associated with higher ischemic stroke hazard. Contrary to type 1 diabetes, type 2 diabetes was not significantly associated with hemorrhagic stroke hazard (HR = 0.99, 95% CI 0.96–1.02). Female sex, lipid-lowering medications, myocardial infarction, heart failure, and higher levels of education and income were significantly associated with lower risk of hemorrhagic stroke. Increased age, use of anticoagulants, and all other comorbidities were significantly associated with increased hemorrhagic stroke hazard.

#### Stratified Analysis by HbA1c Levels

Cox PH regression results stratified by HbA1c levels for type 1 and type 2 diabetes groups and diabetes-free controls are presented in Table 3. For both type 1 and type 2 diabetes, the hazard of ischemic stroke increased with higher HbA1c levels, apart from HbA1c ≤ 42 mmol/mol in type 1 diabetes. Individuals with type 1 diabetes and HbA1c ≤ 42 mmol/mol did not have an increased risk of ischemic stroke compared with diabetes-free controls of the same age and sex from the general population. For hemorrhagic stroke, results differed between type 1 and type 2 diabetes. In type 1 diabetes, no significant association was observed for HbA1c < 52 mmol/mol, but a higher risk was evident at HbA1c levels >52 mmol/mol, with the strongest association and highest risk in the >72 mmol/mol category. In type 2 diabetes, a significantly increased risk of hemorrhagic stroke was observed only for individuals with HbA1c > 72 mmol/mol.

**Table 3:** Cox Regression Results for Type 1 and Type 2 Diabetes Groups and Diabetes-Free Controls Stratified by HbA1c Levels

		n (valid %)	Ischemic stroke		Hemorrhagic stroke	
			HR (95% CI)	*p* Value	HR (95% CI)	*p* Value
Type 1 diabetes						
HbA1c^a^ (mmol/mol)	≤42	2,642 (5.9)	1.54 (0.94–2.50)	0.085	2.20 (0.85–5.70)	0.106
	42–52	6,761 (15.2)	1.93 (1.53–2.45)	<0.001	1.30 (0.74–2.29)	0.362
	52–62	10,273 (23.1)	1.65 (1.41–1.93)	<0.001	1.58 (1.07–2.32)	0.022
	62–72	11,271 (25.4)	2.47 (2.15–2.84)	<0.001	1.81 (1.28–2.56)	<0.001
	>72	13,483 (30.3)	3.83 (3.36–4.36)	<0.001	2.56 (1.82–3.58)	<0.001
Type 2 diabetes						
HbA1c^b^ (mmol/mol)	≤42	109,305 (17.7)	1.13 (1.09–1.16)	<0.001	0.95 (0.88–1.03)	0.246
	42–52	248,213 (40.2)	1.24 (1.22–1.26)	<0.001	0.96 (0.92–1.02)	0.159
	52–62	122,128 (19.8)	1.46 (1.42–1.50)	<0.001	0.98 (0.91–1.05)	0.514
	62–72	60,784 (9.9)	1.66 (1.61–1.72)	<0.001	1.00 (0.90–1.11)	0.967
	>72	76,332 (12.4)	1.82 (1.76–1.89)	<0.001	1.21 (1.10–1.34)	<0.001

Abbreviation: HR = hazard ratio.

All models were adjusted for age, sex, anticoagulants, antiplatelets, antihypertensives, diuretics, lipid-lowering agents, β-blockers/antiarrhythmics, cancer, dementia, alcohol/substance use, hypertension, atrial fibrillation, myocardial infarction, heart failure, TIA, previous stroke, education, and income level.

a Missing values for type 1: 3,290.

b Missing values for type 2: 69,396.

## Discussion

This nationwide observational study investigated the risk of stroke in individuals with type 1 and type 2 diabetes compared with diabetes-free individuals from the general population in Sweden, matched by age and sex. The risk of ischemic stroke was higher for both diabetes types. Individuals with type 1 diabetes also exhibited a higher risk of hemorrhagic stroke compared with diabetes-free controls while type 2 diabetes was significantly associated with risk of hemorrhagic stroke only when HbA1c was higher than 72 mmol/mol.

Decreasing trends in annual incidence rates were observed for both ischemic and hemorrhagic strokes. Similar decreasing trends for both stroke types were found by previous studies from Sweden,^20,21^ which focused on the total population. Similar findings were also reported for high-income countries by the Global Burden of Disease study.^22^ The risk of ischemic stroke was found to be 2.54 times higher in individuals with type 1 diabetes compared with diabetes-free controls in adjusted analysis (2.68 in unadjusted). This association has been shown by previous research^23-25^ while a meta-analysis of cardiovascular disease incidence in type 1 diabetes has estimated the RR of ischemic stroke to be 3.66.^26^ Ischemic stroke risk in type 1 diabetes was, furthermore, associated with glycemic control, with higher risk being observed with increasing HbA1c levels past 42 mmol/mol. Similar results were found by a previous study.^27^ In this study, individuals with type 1 diabetes exhibited an almost 2-fold higher risk of hemorrhagic stroke compared with diabetes-free controls. Hemorrhagic stroke in type 1 diabetes has not been extensively studied previously. The Nurses' Health Study estimated that type 1 diabetes in women was significantly associated with hemorrhagic stroke^24^ while another study found that this association was present in individuals with HbA1c > 52 mmol/mol.^27^ These results are in line with our findings showing that hemorrhagic stroke risk was increased in individuals with type 1 diabetes for HbA1c > 52 mmol/mol, with a 2.6-fold higher risk in the HbA1c > 72 mmol/mol category.

Individuals with type 2 diabetes exhibited a 40% higher adjusted risk of ischemic stroke compared with diabetes-free controls. This finding is in line with findings from previous studies.^9,23,24,28,29^ However, there is high variation in effect sizes across studies regarding the relationship between type 2 diabetes and stroke risk.^9,29^ In this study, the risk of ischemic stroke for type 2 diabetes was 13% higher in the lowest HbA1c category and progressively increased, reaching 82% higher in the highest HbA1c category. Overall, there was no significant association between type 2 diabetes and hemorrhagic stroke in this study. Results from previous research are contradicting^9,29^ with findings ranging from lower risk^10^ and no association^11^ to higher risk in people with diabetes.^12^ However, when stratified by HbA1c levels, a significantly increased hemorrhagic stroke risk was observed only in the highest HbA1c category (>72 mmol/mol), where the risk was 21% higher compared with diabetes-free controls.

An elevated risk of hemorrhagic stroke in individuals with type 1 diabetes, compared with type 2, has been shown previously in women but is not confirmed for men or using population-based data.^24^ Potential explanations include the earlier age at diabetes onset and younger age at hemorrhage in individuals with type 1 diabetes, differentiating them more from the general population.^30^ A large population study found that duration of diabetes and glycemic control are linked to a higher risk of hemorrhagic stroke.^31^ However, the study did not include information on the specific type of diabetes. This study found an association between HbA1c levels and hemorrhagic stroke risk for both diabetes types, with higher risk in type 1 diabetes, evident even at lower HbA1c levels. The younger age at onset in individuals with type 1 diabetes often translates to a longer duration of the disease, which, together with differences in glycemic control, may help explain the increased risk of hemorrhagic stroke observed in this group.

Strengths of this study include the large, population-representative sample size, including nearly 3 million individuals, which, combined with a matched case-control design, enabled robust estimates of stroke risk. The use of multiple registries and the inclusion of various covariates allowed for adjustments across several important factors, enhancing the accuracy of the analysis. Moreover, the high coverage of the NDR, which includes almost all individuals with diabetes in Sweden, minimized selection bias and strengthened the generalizability of the study findings. The distinction between type 1 and type 2 diabetes provided a clearer view of stroke risks for each type, supporting more targeted prevention efforts. The study covered an extensive period of 18 years, providing a long-term view of trends and outcomes. However, only baseline characteristics were used in adjusted analyses, meaning that changes in medications or comorbidities over time were not accounted for. Comorbid conditions and medications may have shifted significantly throughout the study period, which could introduce bias. Cardiovascular risk factors, including laboratory values and smoking status, were available only in the NDR and, therefore, only for individuals with diabetes, limiting their inclusion in adjusted models. Because this is an observational study, it cannot establish causation, but only associations between diabetes and stroke risk. Consequently, unmeasured confounding factors may still influence the observed relationships, and the results should be interpreted with caution. In general, we assert that the findings of this study are likely generalizable, at least to Western countries with publicly funded health care systems, such as that of Sweden.

Both type 1 and type 2 diabetes are associated with an increased risk of ischemic stroke compared with the general population in Sweden, with type 1 diabetes being also associated with heightened risk of hemorrhagic stroke. While there has been a decline in the overall incidence of stroke, the increased relative risk for individuals with diabetes remains significant. These findings highlight the need for targeted stroke prevention strategies in individuals with diabetes. The distinct stroke risk profiles between type 1 and type 2 diabetes emphasize the importance of considering diabetes types when developing clinical interventions and prevention efforts. Further research is needed to better understand the mechanisms behind the increased hemorrhagic stroke risk in type 1 diabetes and to refine stroke prevention strategies for both diabetes types.

## Glossary

BMI: body mass index
BP: blood pressure
eGFR: estimated glomerular filtration rate
HR: hazard ratio
ICD-10: International Classification of Diseases, tenth revision
LDL: low-density lipoprotein
NDR: National Diabetes Register
PH: proportional hazard
RR: risk ratio
